# Supplementary material for: The Effect of Fungal Nutraceutical Supplementation on Postoperative Complications, Inflammatory Factors and Fecal Microbiota in Patients Undergoing Colorectal Cancer Surgery with Curative Intent: A Randomized, Placebo-Controlled, Double-Blind Clinical Trial
Source: Biomedicines. 2025 May 13;13(5):1185. doi: 10.3390/biomedicines13051185 (PMC12108607; doi:10.3390/biomedicines13051185)
Supplement: Supplementary file 1 [file biomedicines-13-01185-s001.zip › biomedicines-3516045-supplementary.pdf]

Supplementary Table S1. Baseline characteristics of the patients undergoing non-robotic surgery.

	Placebo Group (n = 11)	Nutraceutical Group (n = 24)	*p*[1]
Age, (years)	68.0 (61.0-73.0)	67.0 (62.3-72.8)	0.7
Gender, male/female	6/5	10/14	0.7
Tumor location, n (%)			0.7
• Distal colon	6 (54.5)	13 (54.2)	
• Proximal colon	3 (27.3)	9 (37.5)	
• Rectum	2 (18.2)	2 (8.4)	
CRC diagnosis, n (%)			0.7
• positive FIT	5 (45.5)	11 (45.8)	
• Surveillance	4 (36.4)	3 (12.5)	
• Symptoms	2 (18.2)	8 (33.4)	
• Others	0	2 (8.4)	
Duration of symptoms (weeks)	0 (0-13.5)	0 (0-14.8)	0.3
BMI (Kg/m^2)	25.3 (23.6-30.2)	26.0 (23.5-30.2)	0.3
Fat mass (%)	30.8 (24.7-37.7)	31.6 (24.9-40.2)	0.4
Muscle mass (%)	65.7 (59.1-71.1)	64.8 (56.8-71.1)	0.6
Hemoglobin (g/dL)	13.7 (12.4-14.5)	13.7 (12.6-14.3)	0.8
White blood cells ($10^3/\mu L$)	6240 (5235-7070)	6020 (5180-6740)	0.6
Lymphocytes ($10^3/\mu L$)	1290 (1185-1708)	1400 (1115-1743)	0.8
Neutrophils ($10^3/\mu L$)	3620 (2998-4855)	3750 (2900-4928)	0.8
Neutrophil/Lymphocyte ratio	2.81 (2.43-3.30)	2.34 (2.07-2.93)	0.6
Creatinine (mg(dL)	0.8 (0.7-0.9)	0.8 (0.7-0.9)	0.2
Albumin (g/dL)	4.4 (4.3-4.6)	4.4 (4.3-4.5)	0.8
Prothrombin time (seg)	10.7 (10.4-11.1)	10.7 (9.9-11.1)	0.4
IL-6 (pg/mL)	3.3 (2.3-4.8)	3.1 (2.5-4.7)	0.5
IL-10 < 1.6 pg/mL, n (%)	11 (100)	24 (100)	-
TNF-α (pg/mL)	9.0 (7.2-11.4)	9.0 (7.0-11.1)	0.3

[1]Differences between qualitative variables were analyzed with Chi-square test. Differences between quantitative variables were analyzed with Student's t test or Wilcoxon tests if variables did not meet normality. Differences with $p < 0.05$ are considered statistically significant. Continuous variables were presented as medians and interquartile range (IQR). Categorical variables were expressed as frequencies and percentages. FIT: fecal immunochemical test, BMI: body mass index, IL-6: interleukin-6, IL-10 interleukin-10, TNF-α: tumor necrosis factor alpha

Supplementary Table S2. Comparison of nutritional status, quality of life and blood parameters between placebo group and nutraceutical group at the end of follow-up in the subgroup of patients undergoing non-robotic surgery.

	Placebo Group (n = 11)	Nutraceutical Group (n = 24)	p[1]
Creatinine (mg(dL)			
BMI (Kg/m2)	26.2 (23.6-26.2)	26.0 (65.8-30.5)	0.4
Fat mass (%)	30.1 (23.6-30.1)	30.5 (24.5-38.5)	0.1
Muscle mass (%)	66.0 (59.4-66.0)	65.8 (58.4-71.8)	0.1
Quality of life:			
• Physical functioning	100 (90-100)	100 (77.5-100)	0.8
• Physical role,	100 (100-100)	100 (100-100)	0.8
• Emotional role	100 (33.3-100)	100 (66.7-100)	0.5
• Energy/vitality	70 (55-70)	70 (58.3-85)	0.6
• Mental health,	68 (50-68)	80 (61-95)	0.4
• Social functioning	100 (75-100)	100 (87.5-100)	0.4
• Bodily pain	100 (75-100)	100 (70-100)	0.2
• General health perceptions	60 (45-60)	65 (46.3-80)	0.8
Blood parameters:			
• Hemoglobin (g/dL)	13.3 (12.7-13.3)	13.3 (12.7-13.9)	0.8
• White blood cells (103/µL)	6240 (5380-6240)	5990 (4650-7130)	0.9
• Lymphocytes (103/µL)	1170 (953-1558)	1735 (1213-2035)	0.02
• Neutrophils (103/µL)	4005 (2380-4840)	3640 (2318-4278)	0.4
• Neutrophil/Lymphocyte ratio	2.98 (2.34-4.70)	2.02 (1.26-2.62)	0.02
• Creatinine (mg(dL)	0.9 (0.8-0.9)	0.8 (0.8-1.0)	0.4
• Albumin (g/dL)	4.3 (4.2-4.3)	4.2 (4.1-4.4)	0.6
• Prothrombin time (seg),	11.0 (10.5-11.0)	11.0 (10.6-11.7)	0.8
• IL-6 (pg/mL)	3.5 (2.4-3.5)	3.5 (2.7-5.2)	0.4
• IL-10 < 1.6 pg/mL, n (%)	7 (77.8)	18 (90.0)	0.6
• TNF-α (pg/mL)	9.5 (7.8-9.5)	9.2 (7.5-10.5)	0.2

[1]Differences between qualitative variables were analyzed with Chi-square test. Differences between quantitative variables were analyzed with Student's t test or Wilcoxon tests if variables did not meet normality. Differences with $p < 0.05$ are considered statistically significant.

Continuous variables were presented as medians and interquartile range (IQR). Categorical variables were expressed as frequencies and percentages. FIT: fecal immunochemical test, BMI: body mass index, IL-6: interleukin-6, IL-10 interleukin-10, TNF-α: tumor necrosis factor alpha

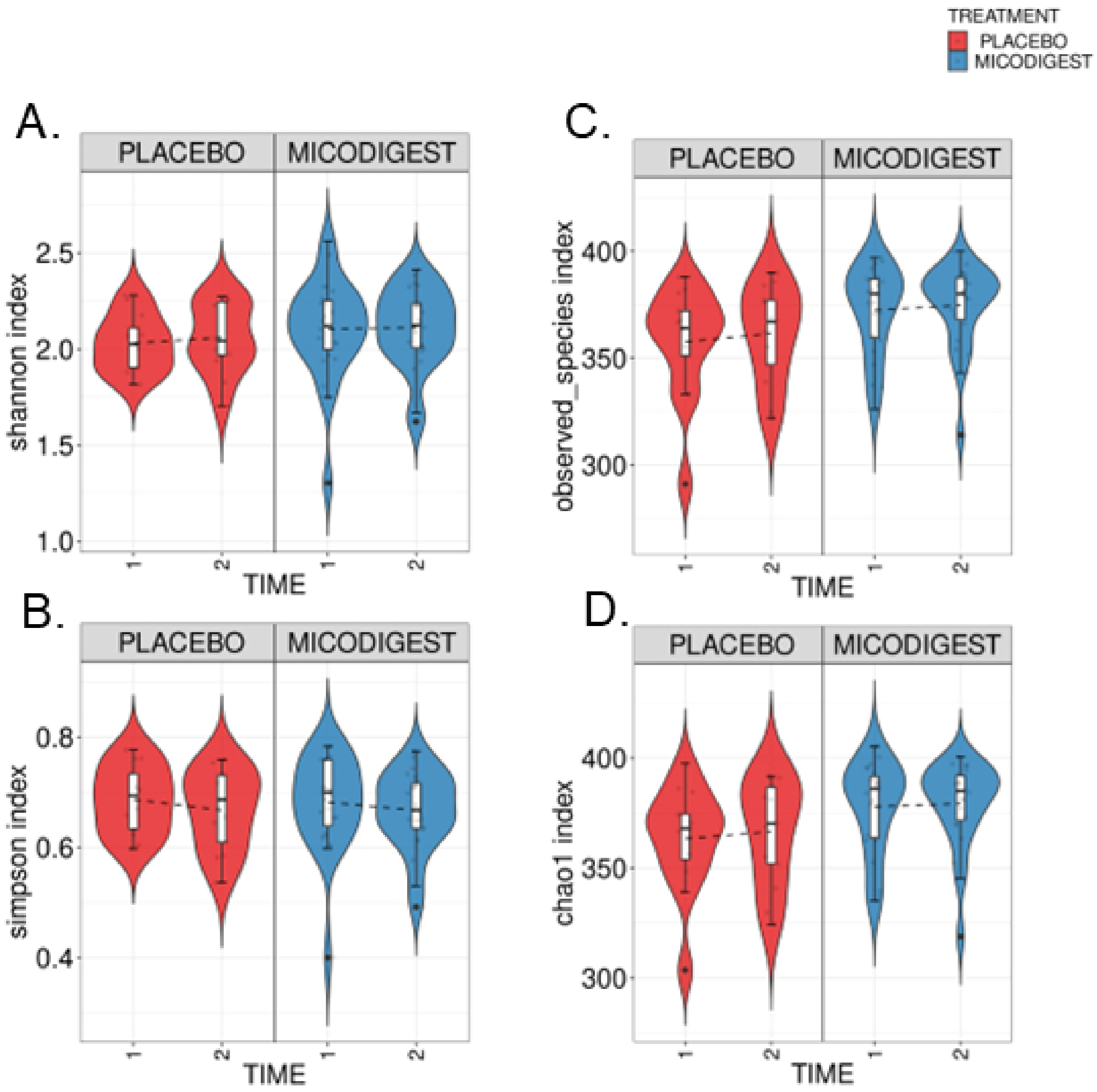

Supplementary Figure S1. Gut bacterial diversity in the placebo group and nutraceutical group (MICODIGEST) of patients at baseline (time point 1) and at the end of the treatment (time point 2). A) Shannon, B) observed species, C) Simpson and D) Chao1 indexes of gut microbial alpha diversity in the fecal samples from the two groups.

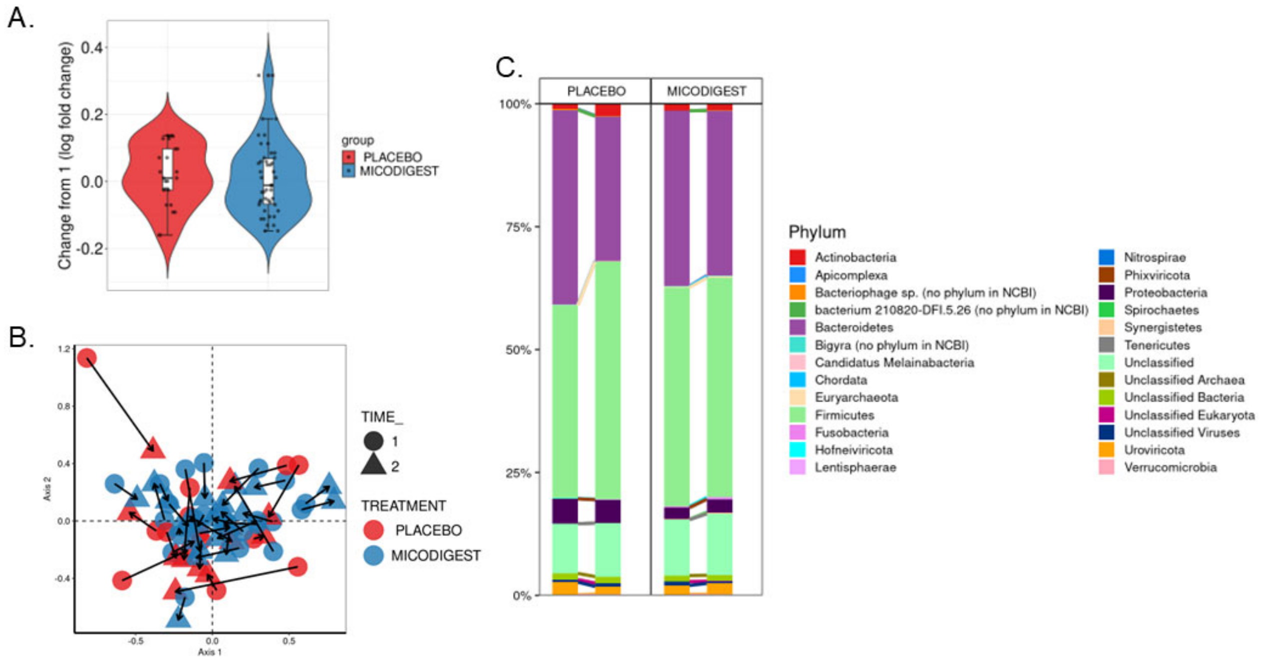

Supplementary Figure S2. Changes in the gut microbiota composition of patients in the placebo and nutraceutical (MICODIGEST) groups at the end of treatment (pretreatment: point time 1, post-treatment: point time 2): (A) Boxplot of patient changes in alpha diversity (Shannon index); (B) PCoA analysis of patient changes in beta diversity based on Jaccard distance; (C) Stacking barplot of taxa changes distribution at the phylum level.

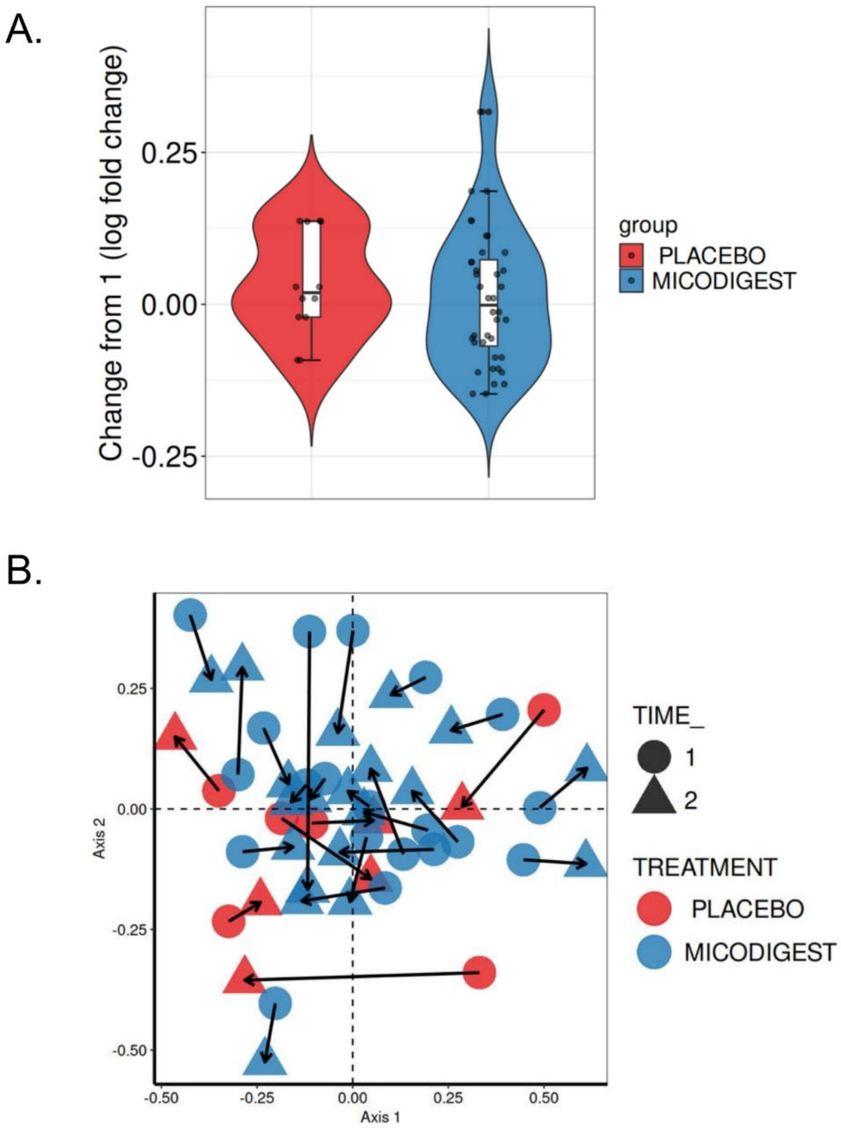

Supplementary Figure S3. Changes in the gut microbiota composition of no-robotic surgery patients in the placebo and nutraceutical (MICODIGEST) groups at the end of treatment. A) Changes in alpha diversity (Shannon index, p = 0.6), B) PCoA analysis of gut microbial beta diversity based on Jaccard distance (p = 0.04).

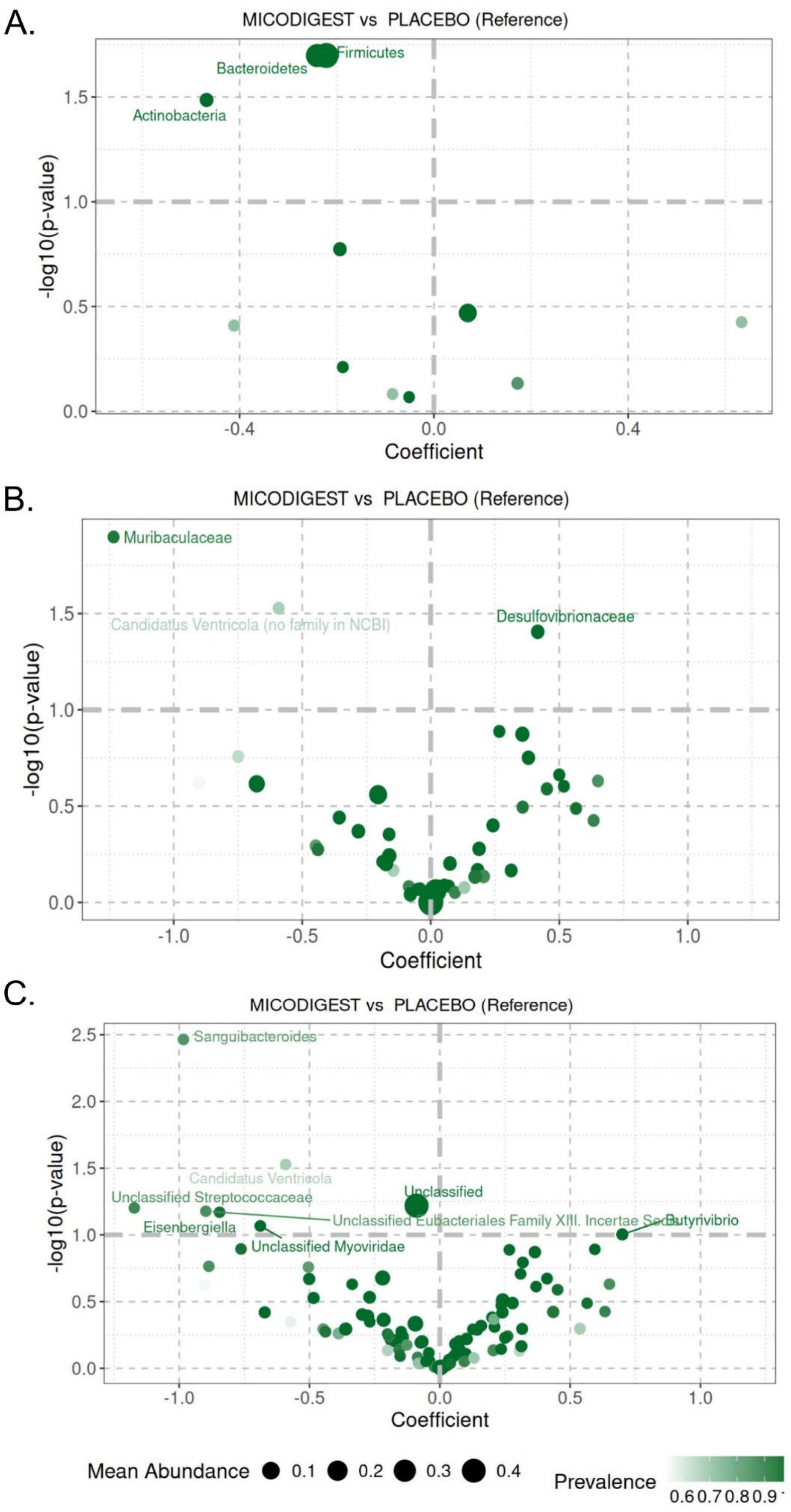

Supplementary Figure S4. Volcano plot showing p-values of linear regression for LinDA at A) phylum, B) family and C) genus level between placebo and nutraceutical (MICODIGEST) groups of no-robotic surgery patients.
